# The Causal Effects of Urban-to-Urban Migration on Left-behind Children’s Well-Being in China

**DOI:** 10.3390/ijerph20054303

**Published:** 2023-02-28

**Authors:** Nan Lu, Wenting Lu, Renxing Chen, Wanzhi Tang

**Affiliations:** 1 School of Economics and Management, Nantong University, Nantong 226019, China; 2 Jiangsu Yangtze River Economic Belt Research Institute, Nantong University, Nantong 226019, China; 3 Shanghai Metropolitan Area Research Institute, Nantong University, Nantong 226019, China; 4 Center for Quality of Life and Public Policy Research, Shandong University, Qingdao 266237, China; 5 School of Political Science and Public Administration, Shandong University, Qingdao 266237, China; 6 Department of Psychology, Faculty of Arts, University of Alberta, Edmonton, AB T6G 2R3, Canada

**Keywords:** migration, left-behind children, China Education Panel Survey (CEPS), propensity score matching (PSM), child well-being

## Abstract

As China’s urbanization process deepens, more and more residents of small and medium-sized cities are moving to large cities, and the number of left-behind children is increasing. In this paper, using data from the China Education Panel Survey (CEPS), a nationally representative survey sample, we examine the well-being of left-behind children with urban household registration at the junior high school level and the causal effects of parental migration on their well-being. Research findings indicate that children who are left behind in urban areas are at a disadvantage in most aspects of their well-being compared to urban non-left-behind children. We examine the determinants of urban household registration for left-behind children. Children in families with lower socioeconomic status, more siblings, and poorer health were more likely to be left behind. In addition, our counterfactual framework reveals that, on average, staying behind negatively impacts the well-being of urban children, based on the propensity score matching (PSM) method. Compared to non-migrant children, left-behind children had significantly lower physical health, mental health, cognitive ability, academic performance, school affiliation, and relationships with their parents.

## 1. Introduction

Families in China can reduce poverty and increase household income by migrating from underdeveloped to developed areas [1]. A common pattern among families is for the parents (one or both) to migrate first and for the children to stay in the outflow area [2]. There are two main reasons. One is the result of the unbalanced development of China’s social and economic structure, with large differences between regions and between urban and rural areas; second, due to a unique household registration system in China (Hukou), migrant children have limited access to education and other social rights [3].

In addition to controlling geographical mobility, the Hukou is also integral to China’s system of social stratification. As a result, Hukou has always been associated with individual rights and benefits in China. In urban areas, Hukou has historically ensured its residents privileges, such as employment, food rationing vouchers, health insurance, housing, and education. Essentially, the Hukou system is responsible for drawing boundaries within Chinese society, thereby resulting in social segregation and social disparities being reproduced. In order to classify Hukou status, there are two main categories, one based on place of residence, and one based on socioeconomic status (often referred to as “agricultural”/“non-agricultural”). The former states that a permanent resident can only register at one address; the latter refers to agricultural and non-agricultural Hukou. It was often more important than the place of registration to determine whether a person was entitled to state-subsidized food and other privileges when the household registration system was established [3]. As China’s economy has developed, the differences in socioeconomic development levels between different regions have gradually increased, exceeding the differences between urban and rural areas [4].

As a result of parents’ migration, two groups of disadvantaged children have been identified: rural left-behind children and urban left-behind children. In China, as the economy grows rapidly and regional disparities widen, some people are migrating from smaller, less developed towns to larger, economically developed ones. It is for the purpose of job opportunities and professional development. There has been a large migration from small towns, small cities, medium-sized cities, and even some large cities in Midwest China to large, developed cities in the eastern region of China. Such factors have contributed to an increase in both the size and proportion of population migration. Urban–urban migrant populations have increased from 46.94 million in 2010 to 82 million in 2020 [5]. There will be a large concentration of urban populations in larger cities, leading to an ever-growing number of urban left-behind children. In 2015, the number of left-behind children in China’s urban areas reached 28.26 million, accounting for 41.1% of all left-behind children in China [6].

The term “well-being” refers to the quality or goodness of an individual’s life existence, referred to as a state of good health, satisfaction, and happiness [7]. UNICEF’s broad definition of child well-being is frequently cited as a reference, i.e., “The true measure of a country’s status is how well it takes care of its children—their health and safety, their material security, their education and socialization, and their sense of being loved, valued and integrated into the family and society into which they were born” [8]. It has been shown that parental migration has conflicting effects on the well-being of left-behind children. Migrating families often benefit from the substantial economic contributions of immigrants [9]. Wealthy families are better able to invest in children and provide a stimulating environment for their children, which benefits their intellectual and emotional development [10]. In this way, remittances from migrants can contribute to the improvement of household living standards, health expenditures, and education for children [11].

Migrants, however, change family structures, and there are a number of consequences for children’s well-being. In the large body of literature, it has been found that left-behind children are more prone to child injury [12,13], accidental injury, and psychological disorders [14,15]. In the absence of parental care, left-behind children are more likely to exhibit cognitive and behavioral difficulties [16,17,18]. Children’s physical and mental health can be negatively impacted by parent–child separation due to a lack of love, care, and parental supervision [19,20,21,22,23,24,25,26]. It is possible for grandparents to provide alternative support for left-behind children, thereby reducing the negative effects of a broken family [27]. However, it has been shown that rural grandparents often lack adequate education and energy to care for their grandchildren [19,28], either spoiling them or not providing them with enough emotional support [29]. Children’s development seems to be compromised by family migration [30,31].

Considering that the above research objects mostly concern rural left-behind children, their conclusions cannot be adapted to urban left-behind children. Studies have shown that there are differences in the impact of migration at different socioeconomic development levels [32,33]. Basic material inputs are most relevant to children’s well-being in under-resourced settings, while they are less relevant in environments with more developed social welfare systems [34]. Urban children have more access to public resources than their rural counterparts. As a result of migration, children who remain in urban areas may not have as much of an impact on their families’ economies. The theory of diminishing marginal utility in economics supports this conclusion. In this case, the key question is whether parental migration of urban left-behind children compensates for the negative effects of parent–child separation.

As mentioned above, most academic studies have focused on rural left-behind children [35,36,37], ignoring urban left-behind children. Furthermore, most studies have examined the effects of migration on single indicators, such as education, psychology, health, and the cognitive ability of migrant children, making it difficult to grasp the impact of migration holistically. Previous empirical studies have mainly used least squares-based regression analyses without considering the net effect of accompanying behaviors on child development.

This paper examines the impact of parental migration on the well-being of left-behind children in urban China using a nationally representative school-based survey (China Education Panel Survey, CEPS). Increasing evidence indicates that migration has a multifaceted and complex impact on children [38]. Children’s well-being was assessed using a variety of indicators, including their school performance, their physical and mental health, and their future aspirations. Immigration processes studied in this paper are common in other social contexts as well, which makes the findings relevant to the broader immigration literature. In conjunction with the findings, policy implications will be discussed.

## 2. Materials and Methods

### 2.1. Data

We analyzed data from the first wave of the China Education Panel Survey (CEPS) in 2014. The China Education Panel Survey (CEPS) is a large-scale, nationally representative, longitudinal survey that starts with two cohorts—the seventh and ninth graders in 2013–2014. A total of 28 county-level units (counties, districts, and cities) were randomly selected as survey sites, and were stratified by average education level and the proportion of mobile population. By documenting the educational processes and transitions students undergo as they progress through various educational stages, the CEPS seeks to explain how education outcomes are influenced by the contexts of families, schools, communities, and social structures. It then further studies how educational outcomes affect people throughout their lives. This data set includes not only basic student information, but also comprehensive family and school information, allowing this study to explore how urban children’s well-being differs in different family configurations.

An approximately 20,000 student sample is selected by random selection from 438 classrooms of 112 schools in mainland China, using a stratified, multistage sampling design with probability proportional to size (PPS). The study is based on a sample of 19,487 middle school students from CEPS, of which 10,687 have rural Hukou and 8800 have non-agricultural hukou. In this study, we will compare the well-being of urban left-behind children with that of local children under the trend of inter-city migration. With rural hukou excluded, the composite question is based on the student’s hukou, the home registration location, and whether parents live together, retaining both urban local children (6057) and urban left-behind children (1470).

### 2.2. Research Design

In order to properly assess the causal impact of parental migration on the well-being of left-behind children in urban areas, we must select appropriate subgroups of children for comparison. Using a cross-classification of parental immigration status and child immigration status, Table 1 presents four types of urban child groups: Type A, non-migrant children of non-migrant parents; Type B, left-behind children of migrant parents; Type C, migrant children of migrant parents; Type D, children who migrate to the city alone, while their parents remain in their home city (this is theoretically possible, but rarely occurs in practice). Type C and D children were not included in our study.

We propose a counterfactual model to investigate the causal impact of parental migration on urban children’s well-being. Our study compared left-behind children of migrant parents (Type B) with urban children of non-migrant parents (Type A). By comparing both groups of children who remain in urban areas, a counterfactual model can be developed to assess the causal impact of parental migration on the well-being of children.

There are several criteria that need to be taken into account when screening these two groups of children. In order to identify urban left-behind children and non-migrant children, we used four variables in the database: type of Hukou, place of household registration, current place of residence, and whether the child is living with their parents. In accordance with this framework, urban left-behind children are children with urban household registration who do not live with their parents and are registered in the same district or county as their current residence. In contrast to left-behind children, non-migrant children live with their parents. In this study, we used non-migrant children as the control group and left-behind children as the intervention group.

### 2.3. Variables

We examined a wide range of child development outcomes in light of the complex and interconnected effects of migration on child well-being. We measured basic indicators, such as health and time use, as well as cognitive and non-cognitive abilities and educational performance, and also covered indicators of interpersonal relationships, especially parent–child relationships. The variable descriptions are presented in Table 2.

Moreover, we include relevant individual, family, and city hierarchy variables in our PSM analysis: (1) variables at the individual level of children (age, gender, birth weight, and whether there is a serious illness before entering primary school); (2) variables of family characteristics (the number of years of education both parents have had, the student’s family economic status prior to primary school, and whether the student is an only child). By examining the hierarchy of cities in which urban children live, this study also captures the types of cities they live in. The detailed description of the control variables is shown in Table 3.

### 2.4. Statistical Model

Classical linear regression models often ignore the endogeneity problem caused by sample selection. In essence, any migration decision is selective [39]. It is often the case that urban families with better economic resources or social capital can overcome institutional obstacles and send their children to better-quality schools when they move. Thus, it is not clear whether left-behind children’s adverse educational outcomes are caused by parental migration or by family resources.

Based on the propensity score matching (PSM) method, we estimated the average intervention effect (ATT) for the intervention group, i.e., the average migration effect of parental migration on urban children. Using notation in the statistical framework of potential outcomes, we assume that $Y_{i}^{T}$ is the outcome of child i under intervention (i.e., parents migrate to another city and children remain at home) and that $Y_{i}^{C}$ is the outcome of the same child without intervention (i.e., parents do not migrate). The ATT can be calculated using the following formula:

$$\mathrm{ATT}=E\left(Y_{i}^{T}-Y_{i}^{C}|D_{i}=1\right)=E\left(Y_{i}^{T}|D_{i}=1\right)-E\left(Y_{i}^{C}|D_{i}=1\right)$$

If children are intervened, Di = 1; otherwise Di = 0. However, if a child is intervened with, it is impossible to observe $Y_{i}^{C}$. If the child received the intervention (i.e., the parents migrated to another city), what would be the child’s well-being compared to a child who did not receive the intervention (i.e., the parents remained in their current city)? Since only one of two outcomes can actually be observed, namely $Y_{i}^{T}$ or $Y_{i}^{C}$, we can only infer the effect of an intervention at the group level rather than at the individual level [40]. For the purpose of inferring the ATT, we formulated a hypothesis that is not necessarily true. As a result, if the non-intervened and intervened children are matched with respect to observable characteristics that have an impact on the intervention, the children do not differ systematically with respect to unobservable characteristics [41]. Assuming that there is a matched control group for each child receiving the intervention based on a set of observed characteristics X, the following condition of independence is met:

$$E\left(Y_{i}^{C}|X,D_{i}=1\right)=E\left(Y_{i}^{C}|X,D_{i}=0\right)=E\left(Y_{i}^{C}|X\right)$$

The ATT can be inferred as follows:

$$\mathrm{ATT}=E\left[Y^{T}|D=1,\mathrm{Pr}(D=1|X)\right]-E\left[Y^{C}|D=0,\;\mathrm{Pr}(D=1|X)\right]$$

where Pr(D=1|X) is the probability of intervention conditional on X. To estimate the effect of parental migration on the well-being of urban left-behind children, we matched left-behind children with non-left-behind children on a number of individual, household, and city-level variables.

We apply PSM on control variables using the user-written Stata package “psmatch2” [42]. We restrict the matched sample to a region of common support, that is, only the matched cases with positive density of propensity scores within both the treatment and control distributions.

## 3. Results

### 3.1. Descriptive Statistics of the Dependent Variables and Matching Variables

Table 2 provides descriptive statistical results on the variables of urban children’s well-being (Appendix A Table A2 presents the results of the *t*-test). The table shows that non-migrant children outperform their left-behind counterparts in most well-being domains, particularly health and cognitive abilities. There was more time spent on housework and leisure activities among the left-behind children than on academic activities. The left-behind children have fewer reliable friends and a dysfunctional relationship with their parents.

**Table 2:** Definitions and descriptive statistics of the dependent variables in the analytical sample of urban children (N = 7527): CEPS 2014.

Dependent Variable	Definition	Left-Behind			Non-Migrant		
		Mean	SD	N	Mean	SD	N
Physical health							
Health	Health level 0 “bad” 1 “good”	0.67 ***	0.47	1456	0.75	0.44	6008
Height	In centimetres	160.80 ***	9.18	1412	162.50	8.95	5918
Weight	In catties (1 catty = 0.5 kg)	98.17 ***	24.14	1328	102.20	23.81	5618
Time use							
Hours per week studying	Duration of time	39.67 ***	36.93	1349	44.19	35.43	5568
Hours per week leisure	Duration of time	50.69 **	40.93	1342	48.84	40.29	5565
Hours per week doing housework	Duration of time	13.61 ***	22.12	1376	10.72	17.78	5711
Cognitive ability	The original total score of the student’s cognitive ability test, the value is [0–22] continuous variable	9.23 ***	3.94	1470	10.30	4.02	6057
Educational performance	The standardized sum scores of the Chinese, Math and English in the mid-term test	206.40 ***	26.85	1429	211.80	26.01	5918
Subjective well-being							
Positive self-perspective	Score of 4 Likert-type items (popularity/happiness/self-confidence/easygoing)	19.32 ***	3.40	1376	19.70	3.41	5764
Attachment to school	Score of 4 Likert-type items (popularity/happiness/self-confidence)	8.54 ***	2.20	1429	9.11	2.21	5916
Depression	Sum score of six-item CES-D	10.79 ***	4.08	1416	10.17	4.19	5872
Educational aspirations	This translates to years of education as 7, 8, 9, 11, 12, 15, 16, 19, and 22 years	15.90 ***	3.64	1392	16.99	3.40	5805
Interpersonal relationship							
Number of good friends	Self-enumerated	11.65	15.35	1407	12.24	16.93	5903
Relationship with father	Self-enumerated	0.66 ***	0.47	1454	0.78	0.41	6034
Relationship with mother	Self-enumerated	0.53 ***	0.50	1432	0.68	0.47	6033

Note: ** *p* < 0.01; *** *p* < 0.001, This is the result of the t-test between the control group and the intervention group for each dependent variable.

Table 3 illustrates that urban left-behind children and non-migrant children have significant differences in the matching variables (Appendix A Table A3 presents the results of the *t*-test). It is more common for urban left-behind children to be seriously ill in preschool, for their parents to have fewer years of education, and for their families to be more economically disadvantaged. The majority of these left-behind children live in small cities. The characteristics of urban left-behind children and non-migrant children are very different, and this imbalance may lead to serious problems with sample “self-selection”. The propensity score matching (PSM) method is used to overcome the issue of self-selection in the sample, in order to determine the net effect of parental migration on urban left-behind children.

**Table 3:** Definitions and descriptive statistics of the matching variables in the analytical sample of urban children (N = 7527): CEPS 2014.

Matching Covariate	Definition	Left-Behind			Non-Migrant		
		Mean	SD	N	Mean	SD	N
Age	A continuous variable whose value is [11–17]	13.58 *	1.22	1428	13.53	1.24	5964
Gender	0 “female”, 1 “male”	0.56 *	0.50	1470	0.51	0.50	6057
Weight at birth	0 “heavy or light”, 1 “normal”	0.76 *	0.43	1255	0.78	0.41	5372
Health in preschool	0 “health”, 1 “poor health”#	0.19 ***	0.39	1215	0.16	0.36	5265
Had sibing (s)	0 “No”, 1 “Yes”	0.43 ***	0.50	1470	0.58	0.49	6057
Father’s education (years)	Continuous variable with value [0–19]	9.92 ***	3.16	1463	11.22	3.47	6042
Mother’s education(years)	Continuous variable with value [0–19]	8.86 ***	3.84	1463	10.43	3.85	6042
Family′s economic state in preschool Family economic in preschool							
Poor	0 “No”, 1 “Yes”	0.23 ***	0.42	1470	0.12	0.33	6057
Medium	0 “No”, 1 “Yes”	0.61 ***	0.49	1470	0.71	0.45	6057
Rich	0 “No”, 1 “Yes”	0.06 **	0.25	1470	0.09	0.29	6057
Not know	0 “No”, 1 “Yes”	0.07	0.26	1470	0.06	0.24	6057
City of residence type							
Province-level municipality	0 “No”, 1 “Yes”	0.02 ***	0.13	1470	0.03	0.17	6057
Provincial city	0 “No”, 1 “Yes”	0.06 ***	0.24	1470	0.13	0.34	6057
Prefecture-level city	0 “No”, 1 “Yes”	0.22 ***	0.41	1470	0.33	0.47	6057
County-level city	0 “No”, 1 “Yes”	0.71 ***	0.46	1470	0.51	0.50	6057

Note: # Children were asked to recall whether they had suffered a major illness before starting elementary school, and we coded those who answered “yes” as 1 (poor health) and those who answered “no” as 0 (healthy). This is the result of the *t*-test between the control group and the intervention group for each matching variable. * *p* < 0.05; ** *p* < 0.01; *** *p* < 0.001.

### 3.2. Matching

Table 4 reports coefficient estimates from logistic models of the propensity to be a left-behind child. We find that boys and girls are equally likely to be left-behind children in cities. There is no evidence that older children are more likely to be left-behind. The children with poor health are more likely to be left behind. The father’s education has a significant negative influence on children’s migration status, because a family’s cultural capital significantly reduces the probability of a child being left-behind. Children with siblings are more likely to be left-behind, probably because older siblings can look after younger ones. A family’s financial situation is also an essential factor. Poor families tend to leave their children at home, as they cannot afford the extra costs of bringing them to cities. Living in a less urbanized county was associated with an increased likelihood of being left-behind. Overall, covariate balance has been improved after matching, because most of the differences between the intervention and control groups before matching have lost their significance (see Table A4).

### 3.3. Effects of Parental Migration

Table 5 presents the estimates of the average treatment effect (ATT) for parental migration after matching. Left-behind children scored significantly lower on the cognitive test and educational performance than non-migrant children. When it comes to patterns of time use, the left-behind children performed more housework and spent more time on leisure activities. There was less positive self-perception and lower educational aspirations in left-behind children, but they were better at regulating their emotions. Nutrition-related outcomes indicated that the left-behind children grew shorter and gained less weight. However, none of these effects are statistically significant. Left-behind children suffered from worse health than the non-migrant children. Left-behind children tended to have less positive relationships with their parents and fewer trusted friends.

## 4. Discussion

As a result of rapid urbanization and economic growth in contemporary China, economic migration has been phenomenal. It is likely to become more prevalent in the near future. While it offers new opportunities to rural migrants, it also poses serious challenges to urban migrants working in cities. Moreover, migrants’ children, especially those left-behind children in their hometowns, are inevitably affected. Leaving left-behind children in their hometowns spares them from the adverse impacts of migration and institutional obstacles in cities. However, separating left-behind children from their parents for a long period of time also negatively impacts their well-being.

There was no significant difference in height and weight between left-behind children and non-migrant children. As a result, the extra economic resources brought back by their migrant parents may not automatically translate into nutritional gains for the children. Meanwhile, the health of left-behind children is significantly inferior to that of non-migrant children. In many cases, the guardians (usually children’s grandparents) are unable to keep a child healthy because they are too old and undereducated. In addition to economic resources, other factors, such as parenting behavior and school quality, may influence the effect of economic resources.

Urban left-behind children performed less well in terms of cognitive ability and academic performance than non-migrant children. However, both the amount of time spent studying and doing housework was not significantly different. The absence of parental guidance may lead to left-behind children being less efficient at learning. As a result of a lack of parental supervision, left-behind children spend more time on leisure activities.

The absence of parents has other negative effects on left-behind children. It has been noted that left-behind children are less likely to have reliable friends and are also likely to have less satisfactory parent–child relationships. Moreover, left-behind children tend to be less attached to school, which is not a positive sign, since weak school attachment is often associated with higher dropout rates. Furthermore, left-behind children suffer from higher rates of depression.

These results indicate that remittances cannot make up for the adverse effects of family separation even if they could benefit left-behind children to some extent. Researchers have found that left-behind children may suffer psychological problems as a result of separation from their parents [28,43]. Nevertheless, since migration can improve living conditions, left-behind children perform better in terms of their physical health, cognitive ability, and academic performance [44,45,46]. Left-behind children generally benefit from rural–urban migration. Urban left-behind children suffer from the parental effects of separation without being able to compensate by improving their objective well-being, resulting in negative effects on mobile urban–urban migrants’ children. What is the reason that parental migration does not benefit the objective well-being of urban left-behind children? Children in urban areas are more likely to have access to schools, teachers, nutritious food, and modern hospitals than those in rural areas. While their parents moved to other cities to work and earn higher incomes, it had a limited impact on the quality of life of the children. In rural areas with limited resources, an increased income is even more beneficial. Although the Chinese government has invested heavily in agricultural areas in recent years, leading to a decrease in the gap between urban and rural areas, rural communities and city communities still have different economic and social environments. Urban–rural heterogeneity must be taken into account when studying China’s problems.

There are several limitations to consider. First, CEPS is a school-based survey of junior high school students, so dropout children are not included. However, the dropout rate for non-migrant and left-behind children is relatively low [47]. Second, most measures of dependent and independent variables are collected through self-reports or proxy-reports by parents, which are, thus, subject to error in reporting. In addition, despite our desire to provide a comprehensive examination of migration’s impact on left-behind children’s well-being, many other interesting questions remain unanswered due to data constraints or space restrictions. For instance, how does the length and timing of children’s or parents’ migration affect their outcomes? What is the difference between the impacts of mother’s migration and father’s migration? What are the differences between boys and girls’ responses to migration? What is the uniqueness of the group of urban left-behind children compared to rural left-behind children? Is there significant heterogeneity within the group of urban left-behind children?

Methodologically, we are still trying to determine the causes of effects rather than focusing on the effects of causes. Inherently, cross-sectional data have the problem of cause and effect occurring at the same time, which can be a problem with either cross-sectional or observational data. Ideally, analysis based on tracking data would examine differences in levels of well-being among the three categories of children who are currently left-behind, those who have experienced being left-behind, and those who have not, as well as the relationship between being left-behind and child well-being over time. We were unable to examine the differences between the three categories of children mentioned above in relation to the temporal relationship between being left-behind and well-being due to data limitations. Research on these issues will contribute to a better understanding of the relationship between parental migration and child well-being. We, therefore, call for more detailed data to be collected and more in-depth research to be performed on this topic. The research results of this paper serve as a foundation for further studies.

## 5. Conclusions

Based on the CEPS 2014, this study uses a propensity-score-matched counterfactual inference model to control for endogeneity issues and preliminarily reveals the impact of the mobility choice of children staying behind on the well-being of urban children. This study revealed the following findings. First, it is not a random decision for urban left-behind children. About 70% of urban left-behind children live in small cities. The health status of individual children, their parents’ education levels, and their families’ circumstances all contribute to children’s left behind status.

Second, after controlling for the selection bias of the sample, urban local children perform significantly better in terms of health status, cognitive abilities, learning ability, and parent–child relationships than urban left-behind children. Consequently, the increased household income of parents after migration is unlikely to cause sudden behavior changes among children, which may negatively affect their wellbeing. As a result of family separation, the well-being of the left-behind children deteriorates.

As China’s urbanization process deepens, the gap between cities is widening. There are, however, few studies on urban–urban migration in China. In this paper, we aim to shift the focus of child well-being research from urban versus rural areas to the differences between cities. Furthermore, the shift in perspective provides some insights into studies relating to migrant populations. In the past, the migrant population was almost synonymous with “migrant workers”, but as it has developed over the past thirty years, it has also become increasingly divided within itself. This paper emphasizes the need to focus on heterogeneity within the migrant population and to distinguish the characteristics of subgroups within the migrant population. China’s urban development policy should be informed by considering the socioeconomic consequences of inter-city differences over the long term, and whether to focus on large central cities or small and medium-sized ones.

## Figures and Tables

**Table 1:** Typology of urban children: CEPS 2014.

Child’s Migration Status		Parental Migration Status	
		No	Yes
Urban child’s migration status (N = 8800)	No	A: Non-migrant	B: Left-behind
		(N = 6057; 68.8 per cent)	(N = 1470; 16.7 per cent)
	Yes	D: Migrant without parent(s)	C: Migrant with parent(s)
		(N = 0; 0 per cent)	(N = 1273; 14.5 per cent)

Note: N refers to the sample size before propensity score matching. The analytical sample is restricted to subgroups A and B, with a total number of 7527 children.

**Table 4:** Estimates of propensity for being left-behind experienced by urban children.

Matching Covariates	Coefficient	SE	*p*-Value
Age	−0.024	0.031	0.426
Gender	0.063	0.071	0.371
Weight at birth	−0.110	0.082	0.182
Health in preschool	0.266 **	0.089	0.003
Had sibing (s)	−0.187 *	0.082	0.022
Father’s education	−0.044 **	0.014	0.001
Mother’s education	−0.009	0.013	0.488
Family economic in preschool (Poor as a reference group)			
Medium	−0.488 **	0.115	0.000
Rich	−0.498 **	0.152	0.001
Not clear	−0.262	0.190	0.169
City type of live (Province-level municipality as the reference group)			
Provincial capital city	−0.084	0.111	0.450
Prefecture-level city	0.011	0.117	0.922
County-level city	0.629 **	0.108	0.000
Constant	−0.212	0.478	0.658
N	5683		

Note: * *p* < 0.05; ** *p* < 0.01.

**Table 5:** Estimates of the average treatment effects on the treated (left-behind children) with non-migrant children as the control group.

Variable name	Left-Behind		Non-Migrant		ATT	SE
	Mean	N	Mean	N		
Physical health						
Health	0.721	724	0.777	3463	−0.055 **	0.020
Height	162.170	724	162.592	3463	−0.422	0.407
Weight	101.499	724	101.529	3463	−0.031	1.135
Time use						
Hours per week studying	46.312	724	44.925	3463	1.388	1.640
Hours per week leisure	50.419	724	46. 561	3463	3.858 *	1.724
Hours per week doing housework	9.650	724	8.553	3463	1.097	0.598
Cognitive ability	10.470	724	10.912	3463	−0.442 *	0.176
Educational performance	210.845	724	214.331	3463	−3.486 **	1.169
Subjective well-being						
Positive self-perspective	19.740	724	19.928	3463	−0.187	0.156
Attachment to school	9.008	724	9.319	3463	−0.311 **	0.099
Depression	10.798	724	10.234	3463	0.564 **	0.207
Educational aspirations	16.894	724	17.112	3463	−0.218	0.152
Interpersonal relationship						
Number of good friends	10.580	724	11.919	3463	−1.339	0.707
Relationship with father	0.682	724	0.797	3463	−0.115 **	0.021
Relationship with mother	0.540	724	0.688	3463	−0.148 **	0.023

Note: * *p* < 0.05; ** *p* < 0.01.

## Data Availability

The raw data used in this study are from the China Education Panel Survey (http://cnsda.ruc.edu.cn/index.php?r=projects/view&id=72810330) (accessed on 3 September 2021), and unrestricted reuse is permitted.

## Appendix A

**Table A1:** Descriptive statistics of the distribution of children in cities (weighted).

Variable Name	Left-Behind	Non-Migrant
City level		
East area	40.60%	55.60%
Central region	33.24%	19.51%
Western region	26.16%	24.89%
Region of city		
Municipality	1.62%	3.13%
Provincial capital city	5.87%	13.09%
Prefecture-level city	21.92%	32.81%
County-level city	70.59%	50.98%
School location		
Central city	35.96%	54.77%
Fringe city	26.67%	22.25%
Rural area	37.38%	22.98%
School ranking		
Medium and below	12.73%	9.93%
Medium	65.12%	57.63%
Excellent	22.15%	32.44%
Family economic in preschool		
Difficulty	28.71%	16.93%
Medium	65.21%	76.29%
Rich	5.37%	6.40%
N	1470	6057

**Table A2:** The *t*-test of the outcome variables in the analytical sample of urban children (N = 7527).

Dependent Variable	Left-Behind	Non-Migrant	*t*-Value	*p*-Value
	Mean	Mean		
Physical health				
Health	0.67	0.75	5.846	<0.001
Height	160.80	162.50	5.312	<0.001
Weight	98.17	102.20	4.354	<0.001
Time use				
Hours per week studying	39.67	44.19	4.171	<0.001
Hours per week leisure	50.69	48.84	−2.692	<0.01
Hours per week doing housework	13.61	10.72	−5.557	<0.001
Cognitive ability	9.23	10.30	8.651	<0.001
Educational performance	206.40	211.80	3.656	<0.001
Subjective well-being				
Positive self-perspective	19.32	19.70	8.139	<0.001
Attachment to school	8.54	9.11	−5.347	<0.001
Depression	10.79	10.17	5.607	<0.001
Educational aspirations	15.90	16.99	8.180	<0.001
Interpersonal relationship				
Number of good friends	11.65	12.24	0.905	>0.05
Relationship with father	0.66	0.78	8.776	<0.001
Relationship with mother	0.53	0.68	11.227	<0.001

**Table A3:** The *t*-test of the matching variables in the analytical sample of urban children (N = 7527).

Matching Covariate	Left-Behind	Non-Migrant	*t*-Value	*p*-Value
	Mean	Mean		
Age	13.58	13.53	−2.226	<0.05
Gender	0.56	0.51	−1.709	<0.05
Weight at birth	0.76	0.78	1.683	<0.05
Health in preschool	0.19	0.16	−3.355	<0.001
Had sibing (s)	0.43	0.58	10.556	<0.001
Father’s education (years)	9.92	11.22	10.804	<0.001
Mother’s education (years)	8.86	10.43	10.766	<0.001
Family economic in preschool				
Poor	0.23	0.12	−11.395	<0.001
Medium	0.61	0.71	5.732	<0.001
Rich	0.06	0.09	2.626	<0.01
Not know	0.07	0.06	−1.055	>0.05
City type of live				
Province-level municipality	0.02	0.03	5.563	<0.001
Provincial city	0.06	0.13	7.354	<0.001
Prefecture-level city	0.22	0.33	4.080	<0.001
County-level city	0.71	0.51	−16.294	<0.001

**Table A4:** Balance test results.

Variable Name	Match Status	Left-Behind	Non-Migrant	*p*-Value
Age	Unmatched	13.386	13.363	0.624
	Matched	13.385	13.411	0.679
Gender	Unmatched	0.502	0.488	0.491
	Matched	0.501	0.491	0.701
Weight at birth	Unmatched	0.770	0.770	0.865
	Matched	0.769	0.798	0.394
Health in preschool	Unmatched	0.212 ***	0.157	0.000
	Matched	0.211	0. 182	0.376
Had sibing (s)	Unmatched	0.611 ***	0.735	0.000
	Matched	0.612	0.625	0.618
Father’s years of education	Unmatched	11.186 ***	12.172	0.000
	Matched	11.201	11.122	0.877
Mother’s years of education	Unmatched	10.592 ***	11.527	0.000
	Matched	10.606	10.544	0.792
Family economic in preschool (Poor as a reference group)				
Medium	Unmatched	0.724 *	0.759	0.045
	Matched	0.725	0.744	0.792
Rich	Unmatched	0.123	0.144	0.134
	Matched	0.123	0.113	0.475
Not clear	Unmatched	0.037	0.042	0.591
	Matched	0.037	0.026	0.239
City type of live (Province-level municipality as the reference group)				
Provincial capital city	Unmatched	0.250 ***	0.345	0.000
	Matched	0.250	0.240	0.666
Prefecture-level city	Unmatched	0.172	0.219	0.005
	Matched	0.173	0.180	0.711
County-level city	Unmatched	0.408 ***	0.233	0.000
	Matched	0.407	0.424	0.513

* *p* < 0.05; *** *p* < 0.001.
